# Special Meeting of the Philadelphia County Dental Society

**Published:** 1889-05

**Authors:** 


### SPECIAL MEETING OF THE PHILADELPHIA COUNTY DENTAL SOCIETY.

HELD AT EARLY'S HALL, WEDNESDAY EVENING, MARCH 27, 1889.

President Frank Basset in the chair.

The President announced a paper entitled:

### A STUDY OF ARSENIOUS ACID.

BY L. ASHLEY FAUGHT, D. D. S., PHILADELPHIA.

Dentistry involving largely a routine mechanical service, its practitioners too often neglect to be thoroughly conversant with the drugs they use. What acquaintance is made with materia medica should be at least sufficiently extended to insure not only safety in use, but ability to cope with the accidental condition of misuse. Not a few of the medicaments employed by dentists are of a poisonous nature, and that to a degree requiring most accurate care in their exhibition; and most prompt and efficient antidotal treatment in case of injurious action. Perhaps none in this respect demands more careful study than arsenious acid. I invite your attention for a few moments this evening to a definite consideration of the aspects of it, which have heretofore been omitted, or but intimated in a very general manner.

"According to the preparation of the arsenic, and the size of the dose, and the manner of its employment, it is either tonic, anti-periodic, pulmonic, detergent, escharotic, or a *vital* irritant (poison)."[1] It is ordinarily used in dentistry for pulp devitalization,

---

[1] Dental Cosmos, vol. XIX, p. 227. Flagg.

and for such purpose has been variously combined in mechanical mixture with other materials. The best formula is the old original one:

℞ Acid arsen....................gr. v
Morph. acet................. gr. x
Creosotum vel. ac. carbol........gr. x
M.

I have never found the other formulæ, subsequently suggested, to possess any additional qualities, of which I did not have the benefit in the one mentioned; while I have, in many, suspicioned the engraftment of undersirable features.

It is not my intention to give my fellow-practitioners instructions in the proper use of arsenious acid; for, if any are in need of lessons, the pages of our text-books are adequate, and to them they are respectfully referred. The province of this paper is to record a few facts gathered from experience, so that any seeking information may not in the future search the professional literature for them in vain, as I have done.

In the first place I desire to suggest that accuracy be used in referring to the drug, naming it as arsenious acid, and not as arsenic, which is so frequently done, for it is an oxide of arsenic, $As_2O_3$; also, that as arsenious acid is poisonous in the highest degree, it is well for everyone using it about the mouth, to acquaint himself with the bulk of the *usual* safe dose, when internally administered. This amount is the $\frac{1}{20}$ of a grain, safety existing between the $\frac{1}{60}$ and $\frac{1}{12}$ of a grain, according to the susceptibility or idiosyncrasy of the patient.

Knowing this quantity definitely, it is positive that systemic trouble cannot supervene, either through local absorption, or in the event of the dressing becoming dislodged and swallowed; for "the quantity of arsenious acid to be employed for devitalization will depend upon the structure and class of the tooth, varying from the $\frac{1}{25}$ to the $\frac{1}{20}$ of a grain."[1]

Reference is here made to these points at the risk of being thought over precise, because it is my belief, sustained by observation, that from our constant contact with the drug there is too often a tendency to carelessness in its use.

So much for the drug itself; I now desire to speak of ways by which arsenious acid is unintentionally introduced into the mouth, and followed not infrequently by pathological conditions.

---

[1] Gorgas' Dental Medicine, p. 130.

The first of these is by uncleanly instrumentation. Care should be taken that the napkin, instruments, vessel containing the medicament, etc., used in making an application, are thoroughly separated from all others, and from contact with the operating tray until cleansed of every trace of the preparation.

I have seen many cases of arsenical ulceration occurring in the mouths of patients in which no arsenious acid had been used; and which were due in all probabilty to the use of instruments that had previously been employed in connection with arsenious acid in another mouth, and returned to the operating case without thorough cleansing; or to the use of instruments, that by virtue of their character, were supposed to have no possible connection with arsenious acid, but to which the drug had been transferred from contact with others contaminated, or with the spot on the tray on which they had rested.

The second way is by the use of rubber dam. Some nine months ago I had an experience in this direction. Quite a number of my patients complained, in immediate succession, of sore mouths. Examination revealed the puzzling condition of unmistakable arsenical ulceration, when I knew that my arsenious acid had not been out of the medicine case for a month.

Recognizing, from previous experience and from the fact that the diseased condition existed only in mouths in which I had been operating with rubber dam, that the cause was the dam, I made application of it to a mouth and produced similar symptoms. The discontinuance of further use of that piece of rubber dam was followed by a cessation of the trouble. I now pemanently ceased to use that make of dam, and fixed for the future upon the product of another standard house.

For nine months I have enjoyed immunity, but within the last week the trouble has recurred. From these and like occurences in past years, the conclusion is drawn that no make of dam can be relied upon, as absolutely safe from arsenical taint, and that ulceration may follow its use at any time when circumstances combine to bring together, a piece so infected (through some irrregularity in its manufacture,) and a susceptible mouth. It is, therefore, to be earnestly recommended that each piece be thoroughly washed with soap and water, before application.

All that is here written, I am well aware, is opposed by the manufacturers of rubber dam, who make an emphatic denial, and say that preparations of arsenic are never used in its production.

As, however, my clinical evidence is indisputable, they certainly must be in error, or the arsenic is unintentionally introduced by contaminate combination with the chemicals used, such as sulphur, soapstone, etc. The latter may possibly be contaminated by the arsenite of calcium, while sulphur is so frequently contaminated by arsenic, that the greatest possibility exists of such introduction by this means.

The third method of accidental introduction occurs through a combination of conditions. I have met more than one with arsenical ulcerations, due to the escape of the medicament upon the removal of old plugs from teeth, in which the pulps had been devitalized at the time of their insertion. In these cases it is evident that the previous operator failed to remove all traces of arsenious acid before dressing and filling the tooth, and it had remained there inert for many years.

The fourth way is through perforations. Teeth have been tapped to relieve the symptoms of incipient abscess which were due to death of the pulp in but one canal of the tooth, and tapped too far, producing an unrecognized perforation. Arsenious acid has then been applied in what was supposed to be a closed cavity —to destroy the vitality of the remaining portion of the pulp, and has escaped through the perforation.

The last way to which I shall refer in this connection is that with which the dental profession is most familiar—the escape of the drug application, by the use of unsuitable coverings to secure it. Writing on the subject of the drug, Dr. Louis Jack says: "Cotton with which wax has been combined, as recommended by Dr. Maynard, cotton partially saturated with sandarach varnish, and gutta-percha are each admirable when the circumstances permit."[1] Prof. James Truman writes, "cover this with a lead cap, and then fill the balance of the cavity with gutta-percha."[2] Notwithstanding the wholesale condemnation which is so frequently made of the use of cotton saturated with sandarach, your essayist has found that his experience agrees with that of Dr. Jack; but he also disagrees with him, with Dr. Truman, and with all others advocating the use of gutta-percha. My experience has been that it is more or less leaky, so that I prefer cement, Dr. Gilbert's temporary stopping, or some similiar preparation.

The influence of arsenious acid, when introduced into the mouth so that it comes in contact with the mucous membrane, is as insidious

[1] American System of Dentistry. Vol. II, p. 173.

[2] American System of Dentistry. Vol. I, p. 902.

as it is destructive. At the moment of escape there is no evidence of the fact. The local effects becoming manifested in from twenty-four to forty-eight hours. There being no pain the destruction of tissues is well progressed before the case comes to the operator's notice. The diseased membrane then presents diagnostic points varying with the interval which has elapsed between the escape of the drug, and the commencment of treatment. If seen early, the membrane has an appearance somewhat as though it had been scratched with a sharp instrument, the wound being red like a piece of raw beef, while the edges and membrane surrounding show simply deepening of the natural color of the gum— a congested condition. At a later period the veins in the surrounding tissues may become prominently developed. If they do not share in the congestion to this extent, the trouble will be of short duration. At the expiration of four days the wounds have the appearance of true ulcers—a deep depressed centre with raised edges—and while not increasing markedly in area, do increase in depth. The action of arsenious acid seeming to destroy, in the first attacked area, by dropping to the bottom of the wound, thus keeping in constant contact with fresh tissue.

Our attention is usually called to arsenical ulcerations by the patient complaining in a similiar strain to this: "My gums feel a little tender since the last operation—they hurt a little in brushing the teeth." They do not complain of pain unless the arsenious acid has access to the periosteum. The pain then is severely acute, with all the symptons of periostitis very much aggravated

The differential diagnosis between arsenical ulceration and rodent ulcers most commonly found in the mouth is not difficult. In the first place, rodent ulcers are generally situated on the soft tissues, inside of the lips, or near the fræmun of the tongue; while arsenical ulcerations have no definite location, may occur any where in the mouth, but are especially prone to be located upon the gums. Then, again, they differ markedly as to appearance. Rodent ulcers are apt to be large and quite painful, while arsenical ulcers are irregular in shape, and like a scratch. Rodent ulcers have a white base, while arsenical ulcers are red, look like raw beef, and are not painful to the touch. With these marked points of difference in appearance, none need err in making a correct diagnosis.

Treatment.—Wherever practical, the soft tissues should be curetted,—scarify freely, wash away the blood and shreded membrane with warm water, and then touch the wound with muriate tincture of iron, or cover with magnesia (Husband's). Then cauterize the wound with carbolic acid, or apply tincture of iodine, and if needed stimulate further in a few days by another application of the last used, "remembering that if we produce inflammation of a part, we check its absorptive power."[1] The attack is usually fully ended in about ten days, and where the treatment here indicated is promptly and thoroughly used, I have never seen the patient much inconvenienced, or the tissue much destroyed;—in other words, no untoward results have followed.

DISCUSSION ON DR. FAUGHT'S PAPER.

*Dr. A. B. Harrower, Philadelphia*—Said that he had only one case of arsenical poisoning through escaping arsenious acid. Did not want another. By mixing the acid with cosmoline the action of the drug was materially slowed. He advised a combination of sulphate of atropia, 1 gr., arsenious acid, 2 grs., and lanoline, 10 parts. He had used such a preparation for the past eighteen months, treating forty-eight cases without any pain. In two instances there had been pain.

*Dr. S. J. Dickey, Philadelphia*—Had had but a limited experience in the use of the drug, has had no trouble, does not believe in its use, depends rather upon careful cleaning and capping.

*Dr. H. Townsand, Philadelphia*—Thought that the sandarach varnish was, in many instances, the cause of the pain. Had had no trouble with ulcerations in the mouth, did not think there need be any if care is used in the application of the drug.

*Dr. Alonzo Boice, Philadelphia*—Never makes an application until he has thoroughly cleansed the cavity, consequently never has any trouble of drug escaping through perforation ; uses the smallest quantity possible—$\frac{1}{500}$ of a grain—which, if it did come in contact with the tissues of the mouth, could do no harm, used freshly ground arsenious acid (Hubbard's), and applied on a very fine probe by puncture. In this manner of using it did not matter much what stopping was used. For his part, generally used cotton saturated with sandarach varnish. Had never had any trouble.

*Dr. C. M. Dixon, Philadelphia*—Probably used more arsenious acid than any one in the city, because his practice ran that way. Had had only one case that gave him any trouble. It was a superior lateral incisor. Had made a second application, requesting patient to return on the second day. She did not do so,

[1] Farquharson's Therapeutics and Materia Medica, p. 152.

but removed the stopping herself, and when next seen had a terribly swollen lip. Treatment consisted in drying lip thoroughly and applying tincture iodine, full strength. Continued treatment daily for four days, when trouble was cured. Had never had any trouble with rubber dam; always washed thoroughly before using. He makes application by touching a small pledget of cotton which had previously been saturated in creosote in dry acid and putting directly on pulp, uses dry cotton to stop with.

*Dr. Chas. E. Hopkins, Philadelphia*—Always cleans the tooth thoroughly and applies on small probe, does not use cotton to make, application, uses Dr. Gilberts stopping to seal cavity.

*Dr. Otto E. Inglis, Philadelphia*—Had had one case of poisoning where application had been made two years previously. It was a bad case and considerable exfoliation occurred necessitating the removal of a sequestrum.

*Dr. Frank Bassett, Philadelphia*—Had seen one case where the process between two molar teeth was destroyed. Always uses as small a quantity as possible, in solutions and stopped with gutta-percha, was afraid sandarach varnish would come out.

In regard to pain following application had never found anything that would satisfactorily control it.

*Dr. Faught* said, in closing that he was somewhat disappointed in the turn the discussion had taken; the paper did not treat of the methods used in applying arsenical applications, but dealt with the question of its misuse, did not think that he was more careless than others in the use of the drug but rather more alert to discover ulceration caused by it. Several bad experiences in early practice had led him to keep a lookout for symptoms indicating trouble, as it was much easier to anticipate trouble than to treat a bad case of ulceration or necrosis. Subject passed.

